# Molecular mechanism of dynamic regulation of the proNGF/NGF ratio in controlling hair follicle cycle transition in liaoning cashmere goats

**DOI:** 10.3389/fcell.2026.1940416

**Published:** 2026-10-02

**Authors:** Bing Luo, Di Han, Meng Sun, Jianwei Wei, Mingqian Li, Shuai Chang, Zhiguo Quan, Ying Wang, Chunqiang Wang, Wei Ma

**Affiliations:** 1 College of Animal Husbandry and Veterinary Medicine, Jinzhou Medical University, Jinzhou, China; 2 Liaoning Key Laboratory of Animal Product Quality and Safety, Jinzhou, China; 3 Liaoning Agricultural Development Service Center, Shenyang, China; 4 Liaoning Cashmere Goat Breeding Farm Co., Ltd., Liaoyang, China

**Keywords:** apoptosis, cashmere goat, hair follicle cycle, nerve growth factor (NGF), signaling balance, transcriptomics

## Abstract

**Background:**

Nerve growth factor (NGF) and its precursor proNGF exert opposing biological functions in cell proliferation and apoptosis through distinct receptor systems—TrkA versus p75NTR/Sortilin. This study aimed to investigate whether dynamic changes in the relative ratio of proNGF to NGF regulate hair follicle cycle transition in Liaoning cashmere goats (Capra hircus) via the dynamic balance of the TrkA/p75NTR signaling axis, and to elucidate the underlying molecular mechanisms

**Methods:**

Skin samples from Liaoning cashmere goats were collected during the telogen (March), anagen (September), and catagen (January) phases. Histological structures of hair follicles were examined via hematoxylin and eosin (H&E) staining. The mRNA and protein expression levels of NGF, proNGF, TrkA, p75NTR, and Sortilin across distinct hair follicle cycle stages were assessed using quantitative real-time PCR (qRT-PCR) and Western blotting. *In vitro*, rat hair follicle stem cells (rHFSCs) and human dermal papilla cells (hDPCs) were employed as cellular models and subjected to 11 different treatment conditions, including NGF alone, proNGF alone, combined treatments with varying proNGF/NGF ratios, and intervention with the TrkA inhibitor K252a. Cell proliferation and apoptosis were evaluated via CCK-8 assay and flow cytometry. Differential ratio groups (control, high-ratio, and low-ratio groups) were selected for transcriptomic sequencing to analyze differentially expressed genes (DEGs) and their enriched signaling pathways.

**Results:**

H&E staining revealed the highest secondary hair follicle activity during the anagen phase. qRT-PCR and Western blot analyses showed that NGF and TrkA expression levels were significantly elevated in anagen (P < 0.05), whereas proNGF, p75NTR, and Sortilin exhibited significantly increased expression in catagen (P < 0.05). The proNGF/NGF protein expression ratio was lowest in anagen (0.56), highest in catagen (1.89), and intermediate in telogen (1.56). CCK-8 assay results indicated that NGF treatment alone significantly promoted the proliferation of both rHFSCs and hDPCs (P < 0.05), an effect that was completely abrogated by K252a; proNGF treatment alone exerted no significant effect on proliferation. At proNGF/NGF ratios of 1.56 and 1.89, cell proliferation rates were significantly lower than those in the NGF-alone group (P < 0.05), whereas at a ratio of 0.56, the proliferation rate showed no significant difference from the NGF-alone group but was significantly higher than that of the control group. Flow cytometry confirmed that proNGF treatment alone and high proNGF/NGF ratios (1.56 and 1.89) significantly induced apoptosis. Transcriptomic sequencing revealed that, in the high-ratio group (proNGF/NGF = 1.89) compared with the low-ratio group (0.56), DEGs were significantly enriched in pathways related to “regulation of apoptotic process” and “steroid hormone biosynthesis,” with notable downregulation of the anti-apoptotic molecules Faim2 and Naip5.

**Conclusion:**

The dynamic changes in the proNGF/NGF ratio serve as a critical molecular determinant governing hair follicle cycle transition in cashmere goats. During the anagen phase, the NGF/TrkA pro-survival signaling predominates, driving follicular growth; conversely, during the catagen phase, the proNGF/p75NTR/Sortilin pro-apoptotic system prevails, inducing follicular regression. The proNGF/NGF ratio may serve as a novel molecular biomarker for hair follicle cycle status. Targeting this balance could offer new therapeutic strategies for regulating cashmere growth in cashmere goats and for treating human hair follicle-related disorders.

## 1 Introduction

Nerve growth factor (NGF) is a member of the neurotrophin family that exerts diverse biological functions across multiple organ systems. Beyond its well-established roles in neuronal survival, differentiation, and synaptic plasticity (Bradshaw et al., 2015), NGF has been shown to participate in a wide range of physiological and pathological processes, including immune regulation (Micera et al., 2007), cardiovascular homeostasis (Caporali and Emanueli, 2009), tissue repair and wound healing (Aloe et al., 2015), and stem cell maintenance (Zhang, 2026). The biological actions of NGF are mediated through two distinct receptors: the high-affinity tyrosine kinase receptor TrkA and the low-affinity p75 neurotrophin receptor (p75NTR). While TrkA activation typically promotes cell survival and proliferation, p75NTR signaling can induce either apoptosis or survival depending on the cellular context and the presence of co-receptors such as Sortilin (Bradshaw et al., 2015). The complexity of NGF signaling is further amplified by the existence of its precursor, proNGF, which exhibits opposing biological functions through preferential binding to the p75NTR/Sortilin complex (Nykjaer et al., 2026). In the skin, the NGF/proNGF system has emerged as a critical regulator of hair follicle biology, although the precise molecular mechanisms governing its stage-specific actions remain incompletely understood.

Hair follicles are unique miniature organs within mammalian skin, possessing the capacity for lifelong cyclical regeneration. The hair cycle comprises three distinct phases: anagen (active growth phase, characterized by rapid cell proliferation and fiber production), catagen (regression phase, marked by apoptosis-driven follicular involution), and telogen (resting phase, during which the follicle is quiescent before re-entering anagen). The hair cycle comprises three distinct phases: anagen, catagen, and telogen, the precise regulation of which depends on the coordinated interplay between hair follicle cells and multiple signaling pathways within the microenvironment (Liu et al., 2026). In animals used for agricultural and industrial production, such as cashmere goats, the cyclic development of secondary hair follicles directly determines both the yield and quality of cashmere. Secondary hair follicles are fine, wool-producing follicles that arise from the primary follicles and are responsible for the production of the cashmere fiber. Unlike primary follicles, which produce coarse guard hairs, secondary follicles undergo seasonal cyclic regeneration and determine the softness and commercial value of cashmere. Therefore, an in-depth understanding of the molecular mechanisms governing hair follicle cycle regulation holds significant theoretical importance and practical application value.

Nerve growth factor (NGF), is a member of the neurotrophin family, is classically recognized to signal through two receptors: p75NTR and the tyrosine kinase receptor TrkA. The prevailing paradigm holds that when TrkA is present on the cell membrane, NGF binding to TrkA activates pro-survival and pro-proliferative signaling cascades (including PI3K-Akt, TRAF6-NF-κB, and Ras-MAPK pathways), thereby promoting cell proliferation. Conversely, NGF binding to p75NTR exerts pro-apoptotic effects on neurons, myocytes, and lymphocytes. However, accumulating evidence indicates that even in cells co-expressing both TrkA and p75NTR, apoptotic signals can still be triggered, counteracting the growth-promoting signals induced by NGF (Kropf et al., 2023). This phenomenon is attributed to the actions of the nerve growth factor precursor (proNGF) and Sortilin—the third receptor for NGF—which initiate intracellular apoptotic signaling (Arnett et al., 2007). Although NGF is present at very low concentrations *in vivo*, it is intimately associated with the growth and development of target cells. The organism initially synthesizes and secretes proNGF, which is subsequently cleaved by proteases into mature NGF. Both NGF and proNGF modulate cell fate—survival versus death—through their differential binding to the high-affinity receptor TrkA and the low-affinity receptor p75NTR.

In recent years, the role of the NGF/proNGF system in hair follicle cycle regulation has garnered increasing attention. Studies by Peters et al. (2006) in mice provided important insights into the stage-specific roles of the NGF system in hair follicle cycling. In their study, hair induction—referring to the experimental initiation of a new anagen phase in depilated or plucked mouse skin, a well-established model for studying hair cycle activation—was used to synchronize follicular regeneration. They found that NGF/proNGF mRNA levels peaked on day 1 following hair induction, whereas protein levels reached their maximum during catagen, suggesting active post-transcriptional regulation. Immunohistochemical analyses revealed that NGF and its high-affinity receptor TrkA were predominantly localized in the epidermis and the proliferative compartments of hair follicles, whereas proNGF was highly expressed in the differentiated inner root sheath and in p75NTR-positive degenerating epithelial strands during catagen. To investigate functional effects, the authors used 7 S NGF—a naturally occurring complex isolated from mouse submandibular glands that contains a mixture of mature NGF, proNGF, and associated proteins (such as zinc and proteases)—which has been widely used in historical studies of NGF biology. They demonstrated that 7S NGF promoted anagen progression in early anagen skin organ cultures, yet induced catagen advancement in late anagen skin (Wang et al., 2019). These findings suggest that the NGF system exerts context-dependent and stage-specific effects on hair follicle cycling, with NGF/TrkA signaling promoting anagen maintenance and proNGF/p75NTR signaling facilitating catagen entry.

Despite the important foundation established by the aforementioned studies, several key scientific questions remain to be addressed. First, previous investigations have largely focused on the absolute expression levels of NGF or proNGF as individual molecules, while overlooking the relative ratio of the two—a parameter of greater biological significance. Given that NGF and proNGF originate from the same precursor gene (NGF), differences in post-translational processing efficiency determine the ultimate ratio of NGF to proNGF, which in turn dictates the direction of signal output: a bias toward TrkA favors pro-survival signaling, whereas a bias toward p75NTR promotes pro-apoptotic signaling. Therefore, the proNGF/NGF ratio itself may represent a more critical regulatory node than the absolute expression level of either molecule alone (Xie et al., 2024). Second, current understanding of the NGF/proNGF system in hair follicle cycling is derived primarily from studies in murine and human hair follicles, with systematic investigations in cashmere goats—an economically significant livestock species—remaining considerably limited. Third, the molecular mechanisms by which changes in the proNGF/NGF ratio influence hair follicle cell fate through downstream transcriptomic reprogramming remain largely elusive.

In this study, only female Liaoning cashmere goats were selected for sample collection. This choice was based on two considerations: first, female goats are predominantly used in cashmere production due to their superior fiber fineness and uniformity compared to males; second, the use of females minimized confounding effects from androgen-driven seasonal variations in hair follicle cycling, allowing for a more focused assessment of the NGF/proNGF system without interference from sex hormone fluctuations.

For *in vitro* functional validation, we employed rat hair follicle stem cells (rHFSCs) and human dermal papilla cells (hDPCs) as cellular models. These 2 cell types represent the epithelial (stem cell) and mesenchymal (dermal papilla) compartments of the hair follicle, respectively, and their reciprocal interactions are essential for hair cycle regulation. Both are commercially available, well-characterized immortalized cell lines that have been widely used in hair biology research, allowing for reproducible and controllable *in vitro* experiments. While we acknowledge that primary cashmere goat cells would be the ideal choice for species-matched validation, the use of these established cell lines provides a reliable platform for initial mechanistic exploration of the conserved NGF/proNGF signaling pathways, and offers a foundation for future studies using goat-specific primary cells.

Against this background, the present study employed Liaoning cashmere goats as the experimental model and systematically examined the expression dynamics of NGF, proNGF, and their receptors TrkA, p75NTR, and Sortilin across distinct hair follicle cycle stages. For the first time, we calculated and analyzed the cycle-dependent dynamic changes in the proNGF/NGF ratio. On this basis, using *in vitro* models of rat hair follicle stem cells and human dermal papilla cells, we precisely manipulated the proNGF/NGF ratio in combination with intervention using the TrkA-specific inhibitor K252a to validate the functional effects of this ratio on cell proliferation and apoptosis. Finally, transcriptomic sequencing was employed to delineate the differential gene expression profiles and enriched pathways under varying proNGF/NGF ratios, thereby providing systematic evidence to elucidate the molecular mechanisms by which the dynamic balance of the proNGF/NGF ratio governs hair follicle cycle transition.

## 2 Materials and methods

### 2.1 Ethics statement

All methods and procedures were performed in accordance with the relevant guidelines established by the Ministry of Agriculture of the People’s Republic of China. The ethics approval for this study was obtained from the Ethics Committee of Jinzhou Medical University (JZMULL2026130).

### 2.2 Experimental animals and sample collection

Skin tissues were collected from six adult female Liaoning cashmere goats (n = 6) from the core flock of Liaoning Cashmere Goats maintained at the Liaoning Academy of Animal Science. All experimental animals were raised under identical feeding conditions and growth environments. Skin samples were collected from the dorsal region along the upper margin of the scapula using a biopsy punch (diameter 1 cm) during the telogen (March), anagen (September), and catagen (January) phases. From each goat, skin samples were collected during telogen (March), anagen (September), and catagen (January), resulting in three biopsies per animal. Each biopsy was immediately divided into two portions under sterile conditions. One portion was fixed in 4% paraformaldehyde for histological analysis (H&E staining). The other portion was snap-frozen in liquid nitrogen and subsequently pulverized under cryogenic conditions; the resulting powder was then equally divided into two aliquots: one for total RNA extraction (for qRT-PCR) and the other for total protein extraction (for Western blotting). This approach ensured that both RNA and protein were derived from the same tissue sample, enabling direct comparison of transcript and protein expression levels.

### 2.3 H&E staining of skin tissues

Skin tissue samples fixed in formalin were processed using an automatic tissue dehydrator (KRD-TA, China), embedded in paraffin using a paraffin embedding machine (KRD-LB2, China), and subsequently sectioned into 5-μm-thick slices using a rotary microtome (HistoCore BIOCUT, China). The sections were stained according to the classical hematoxylin and eosin (H&E) staining protocol.

### 2.4 mRNA detection of target genes

#### 2.4.1 Total RNA extraction from skin tissues and cDNA synthesis

Immediately after collection, skin tissue samples were placed in pre-cooled (4 °C) RNA stabilization solution and homogenized using a low-temperature grinder for 30 s. Total RNA was then extracted using a modified Trizol-based method. cDNA synthesis was performed using a reverse transcription kit, with a total reaction volume of 20 μL (Table 1). The reaction conditions were as follows: 37 °C for 2 min, 56 °C for 15 min, and 85 °C for 2 min.

**TABLE 1:** Reverse transcription reaction system.

Component	Volume
RNA template	3 μL
StarScript pro all-in-one RT mix	1 μL
5×StarScript pro all-in-one RT buffer	4 μL
Nuclease-free water	12 μL

#### 2.4.2 Quantitative real-time PCR (qRT-PCR)

##### 2.4.2.1 Primer Design and Synthesis

The sequence information for goat NGF, proNGF, TrkA, p75NTR, Sortilin, and the reference gene GAPDH was retrieved from the GenBank database. Primers were designed using the NCBI database and synthesized by Shanghai Sangon Biotech Co., Ltd. (Table 2).

**TABLE 2:** Primer information for NGF and other target genes, and GAPDH.

Gene	Primer sequence (5′→3′)	Product size (bp)
NGF	F′: AAGCGGCGACTGCGTTCA R′: TCCCAGCACCATCACCTCC	249
proNGF	F′: CATGCTGGACCCAAGCTCAG R′: GGACATTACGCTATGCACCTTA	281
TrkA	F′: TCGGCGATTTCGGTATG R′: GTGGTGAACTTGCGGTAGAG	121
P75^NTR^	F′: GGGGACCAAACAACATCATC R′: AAGGGCTCATGACCACAGTC	172
Sortilin	F′: TCGCCAGTGGACACTAACAG R′: AGGAGAGAACCTGCTGGTGA	189
GAPDH	F′: GGGTCATCATCTCTGCACCT R′: ACAGTCTTCTGGGTGGCAGT	211

##### 2.4.2.2 Quantitative Real-Time PCR

Quantitative real-time PCR was performed using SGExcel FastSYBR qPCR Master Mix (provided by Shanghai Sangon Biotech Co., Ltd.). The GAPDH gene was used as the internal reference gene, and each sample was analyzed in triplicate. The reaction system is shown in Table 3. The amplification conditions were as follows: initial denaturation at 95 °C for 3 min, followed by 40 cycles of denaturation at 95 °C for 5 s and annealing at 60 °C for 20 s.

**TABLE 3:** Quantitative real-time PCR reaction system.

Reagent	Volume
2×SGExcel FastSYBR mixture	10 μL
Forward Primer, 10 μM	0.6 μL
Reverse Primer, 10 μM	0.6 μL
Template DNA	3 μL
100×ROX reference dye	0.2 μL
RNase-free ddH_2_O	6.6 μL

### 2.5 Detection of target proteins

Total protein was extracted from skin tissues using a whole-protein extraction kit (Beijing Solarbio Science and Technology Co., Ltd.). Protein samples were separated by SDS-PAGE using 10% separating gel and 5% stacking gel. Electrophoresis was performed at an initial voltage of 80 V, which was adjusted to 120 V after the bromophenol blue dye front entered the separating gel. Following electrophoresis, the gel was equilibrated in transfer buffer (containing 20% methanol) for 15 min and then transferred onto membranes at a constant current of 0.12 A for 30 min. The membranes were blocked with blocking buffer at room temperature for 2 h on an orbital shaker, followed by overnight incubation with primary antibodies at 4 °C (primary antibodies, 1:1000; β-actin, 1:10,000). After washing with TBST, the membranes were incubated with secondary antibodies (1:5000) at room temperature for 1 h. Protein bands were visualized using an enhanced chemiluminescence (ECL) detection reagent.

### 2.6 Cell culture

Rat hair follicle stem cells (rHFSCs) and human dermal papilla cells (hDPCs) were purchased from Shanghai iCell Biotechnology Co., Ltd., both of which were immortalized cell lines. All cells were cultured in specialized media supplemented with 10% fetal bovine serum (FBS) and 1% penicillin-streptomycin at 37 °C in a humidified incubator with 5% CO_2_. Upon reaching 80%–90% confluence, the cells were passaged using 0.25% trypsin-EDTA. All experiments were performed using cells in the logarithmic growth phase, with passage numbers ranging from 5 to 10.

### 2.7 Experimental grouping

In this study, rat hair follicle stem cells (rHFSCs) were employed as a model for follicular epithelial cells, and human dermal papilla cells (hDPCs) were used as a representative of follicular mesenchymal cells. The cells were divided into the following treatment groups:

1. Control group: cultured in basal medium without growth factors.
2. NGF treatment group: cultured in medium supplemented with recombinant NGF protein at a specified concentration.
3. proNGF treatment group: cultured in medium supplemented with recombinant proNGF protein at a specified concentration.
4. proNGF/NGF combined treatment groups: cultured in medium supplemented with recombinant proNGF and NGF proteins at specific ratios.
5. Inhibitor groups: to verify signaling pathway specificity, combinations including the TrkA inhibitor K252a were added.

In total, 11 experimental groups were established in this section: CON (control), NGF, proNGF, NGF + K252a, proNGF + K252a, proNGF/NGF = 1.56, proNGF/NGF = 0.56, proNGF/NGF = 1.89, proNGF/NGF = 1.56 + K252a, proNGF/NGF = 0.56 + K252a, and proNGF/NGF = 1.89 + K252a. NGF and proNGF were each used at a concentration of 75 ng/mL, and the TrkA inhibitor K252a was used at 100 nM.

### 2.8 Cell proliferation assay

Rat hair follicle stem cells (rHFSCs) and human dermal papilla cells (hDPCs) were seeded into 96-well plates at a density of 5 × 10^3^ cells per well. After 24 h of culture, the medium was replaced with medium containing the respective treatment factors. Following an additional 24 h of incubation, 10 μL of CCK-8 reagent was added to each well, and the plates were incubated for 2 h. Absorbance was measured at 450 nm using a microplate reader. Cell viability was calculated as follows: Viability (%) = (OD_treatment_ − OD_blank_)/(OD_control_ − OD_blank_) × 100%. Each experiment was performed in triplicate.

### 2.9 Cell apoptosis detection

rHFSCs were seeded into 6-well plates at a density of 2 × 10^5^ cells per well. After 24 h of culture, the medium was replaced with medium containing the respective treatment factors, and the cells were cultured for an additional 24 h. The culture supernatant and detached cells following trypsinization were collected, centrifuged, and washed with PBS. The cells were then stained with Annexin-V-PE/7-AAD and incubated at room temperature in the dark for 15 min, followed by flow cytometric analysis. The total apoptosis rate was calculated as the sum of early apoptotic (Annexin V^+^/7-AAD^-^) and late apoptotic (Annexin V^+^/7-AAD^+^) cell populations.

### 2.10 Transcriptomic sequencing

Based on the results of the aforementioned cell proliferation and apoptosis assays, the high-ratio group (proNGF/NGF = 1.89), the low-ratio group (proNGF/NGF = 0.56), and the control group (CON) were selected for transcriptomic sequencing, with four biological replicates per group. After 24 h of treatment, total RNA was extracted from rat hair follicle stem cells using the Trizol method. RNA concentration and purity were assessed using a NanoDrop ND-1000 spectrophotometer, and RNA integrity was evaluated using a Bioanalyzer 2100 (RIN value >7.0). Qualified samples were used for subsequent library construction. Sequencing was performed on the Illumina PE150 platform. Raw reads were processed to remove adapters and low-quality sequences to obtain clean reads, which were then aligned to the rat reference genome (GCF_015227675.2_mRatBN7.2) using Hisat2. Differential expression analysis was conducted using edgeR, with significance thresholds set at |log_2_FC| ≥ 1 and q < 0.05. Gene Ontology (GO) functional enrichment and Kyoto Encyclopedia of Genes and Genomes (KEGG) pathway enrichment analyses of differentially expressed genes (DEGs) were performed using the clusterProfiler software, with a corrected P < 0.05 considered statistically significant. Differentially expressed genes, including Faim2 and Naip5, were randomly selected for qPCR validation to confirm the consistency of expression trends.

### 2.11 Statistical analysis

All experiments in this study were performed in at least triplicate. Data were analyzed using one-way analysis of variance (ANOVA) with SPSS 26.0 software, and a P-value <0.05 was considered statistically significant. ImageJ and GraphPad Prism software were used for data visualization and graphical representation.

## 3 Results

### 3.1 Histological observation of hair follicles

Hematoxylin and eosin (H&E) staining was performed to examine the histological changes of hair follicles across the three phases of the hair cycle in Liaoning cashmere goats. The staining results revealed that the structural organization of hair follicle groups remained clearly discernible throughout all three phases of the hair cycle in cashmere goat skin. Notably, compared with the catagen and telogen phases, the anagen phase exhibited higher secondary hair follicle activity, a larger follicle-to-skin ratio, and more prominent structural characteristics of follicle groups (Figure 1).

**FIGURE 1:**
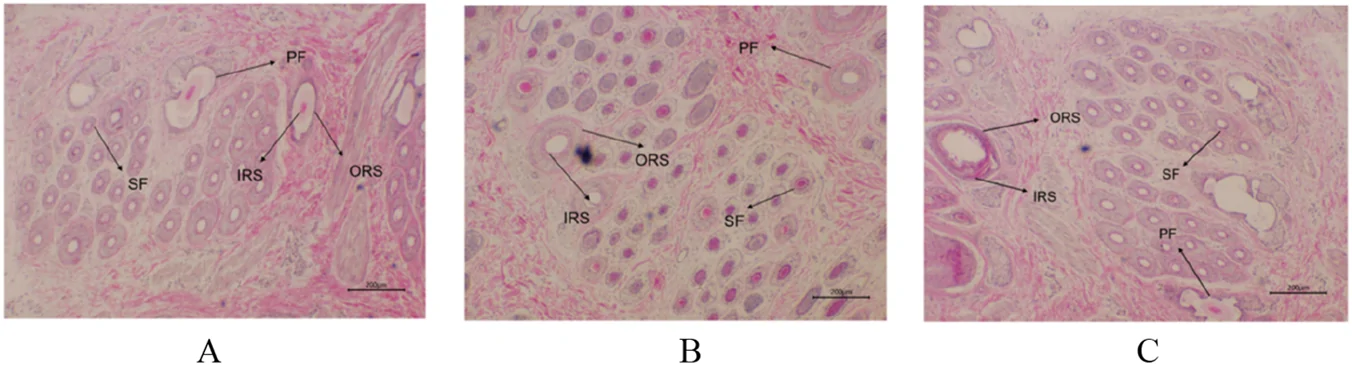
Histological observation of skin hair follicles across different phases of the hair cycle. Note: **(A)** Cross-section of skin hair follicle in telogen; **(B)** Cross-section of skin hair follicle in anagen; **(C)** Cross-section of skin hair follicle in catagen. PF: primary hair follicle; SF: secondary hair follicle; IRS: inner root sheath; ORS: outer root sheath.

### 3.2 Total RNA extraction and quantitative real-time PCR detection of target genes

Total RNA was extracted from skin samples using the Trizol method. Ultramicrospectrophotometric measurements showed that the OD_260_/OD_280_ ratios of total RNA ranged from 1.9 to 2.0, meeting the quality requirements for RT-PCR analysis.

Quantitative real-time PCR results demonstrated that the NGF system exhibited stage-specific expression patterns throughout the hair follicle cycle in Liaoning cashmere goats. In anagen samples, the mRNA expression levels of NGF and proNGF were significantly elevated compared with those in telogen and catagen (P < 0.05), and the expression trend of its high-affinity receptor TrkA was consistent with that of its ligand. In catagen, the mRNA expression levels of the receptor complex components p75NTR and Sortilin were significantly increased (Figures 2A–E).

**FIGURE 2:**
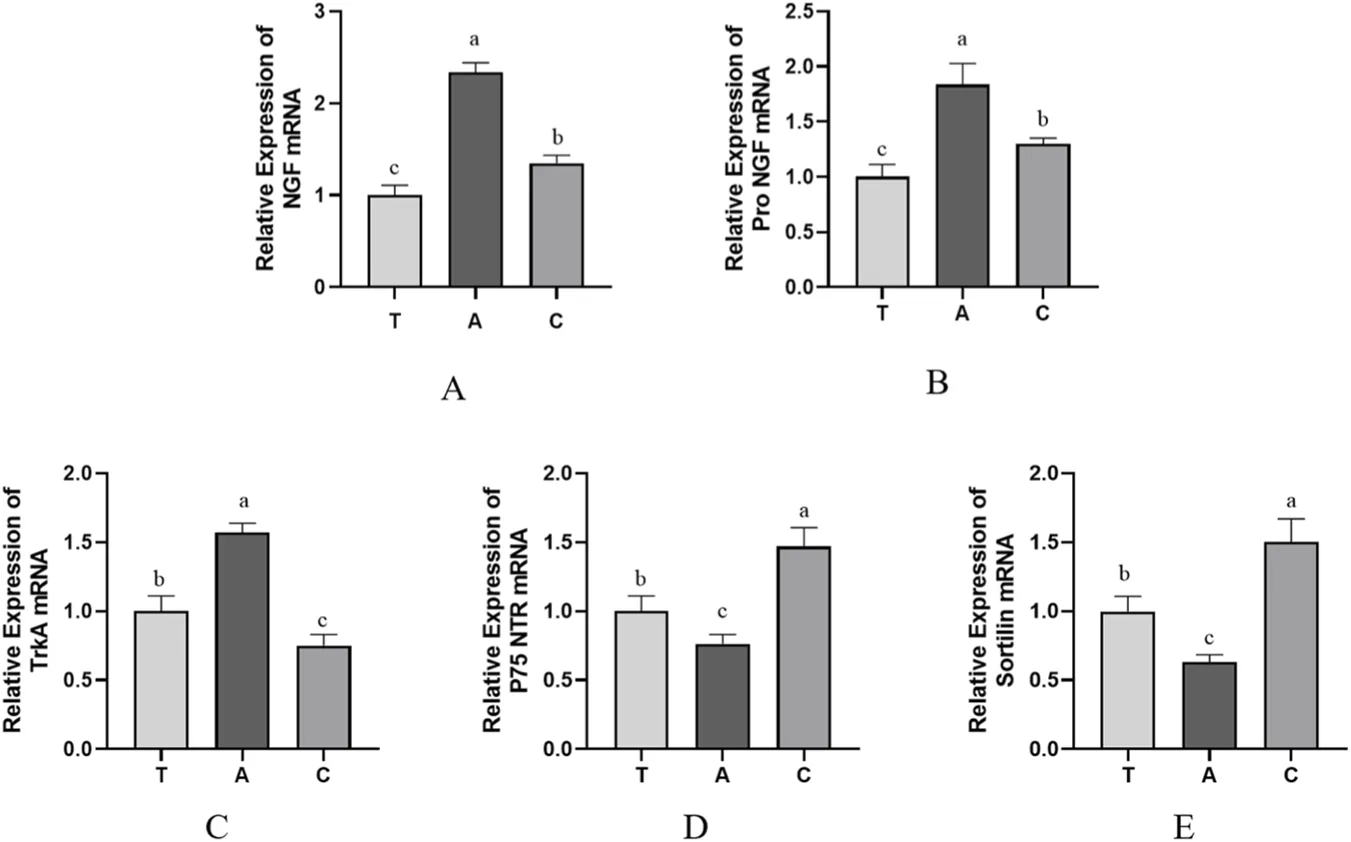
Quantitative real-time PCR analysis of target genes in cashmere goat skin tissues. Note: T, telogen; A, anagen; C, catagen. Panels **(A–E)** represent the relative mRNA expression levels of NGF, proNGF, TrkA, p75NTR, and Sortilin, respectively. Columns without common superscript letters indicate significant differences (*P* < 0.05).

### 3.3 Detection of target proteins

To systematically examine the protein expression levels of proNGF/NGF and their receptor system across distinct phases of the hair follicle cycle in cashmere goats, immunoblotting analysis was performed for NGF, proNGF, TrkA, p75NTR, and Sortilin (Figure 3A). The results demonstrated that the protein expression level of NGF was significantly higher in anagen than in telogen and catagen (*P* < 0.05) (Figure 3B), and the expression pattern of TrkA was consistent with that of NGF, showing significantly elevated expression in anagen (*P* < 0.05) (Figure 3D). These findings further confirmed, at the protein level, the coordinated upregulation of NGF and its high-affinity receptor TrkA during anagen. On the other hand, the protein expression levels of proNGF and its receptors p75NTR and Sortilin all peaked in catagen, significantly surpassing those observed in anagen and telogen (*P* < 0.05) (Figures 3C–F). Collectively, these data reveal a distinct stage-specific and antagonistic expression pattern at the protein level between the pro-survival system (NGF, TrkA) and the pro-apoptotic system (proNGF, p75NTR, Sortilin) throughout the hair follicle cycle in cashmere goats.

**FIGURE 3:**
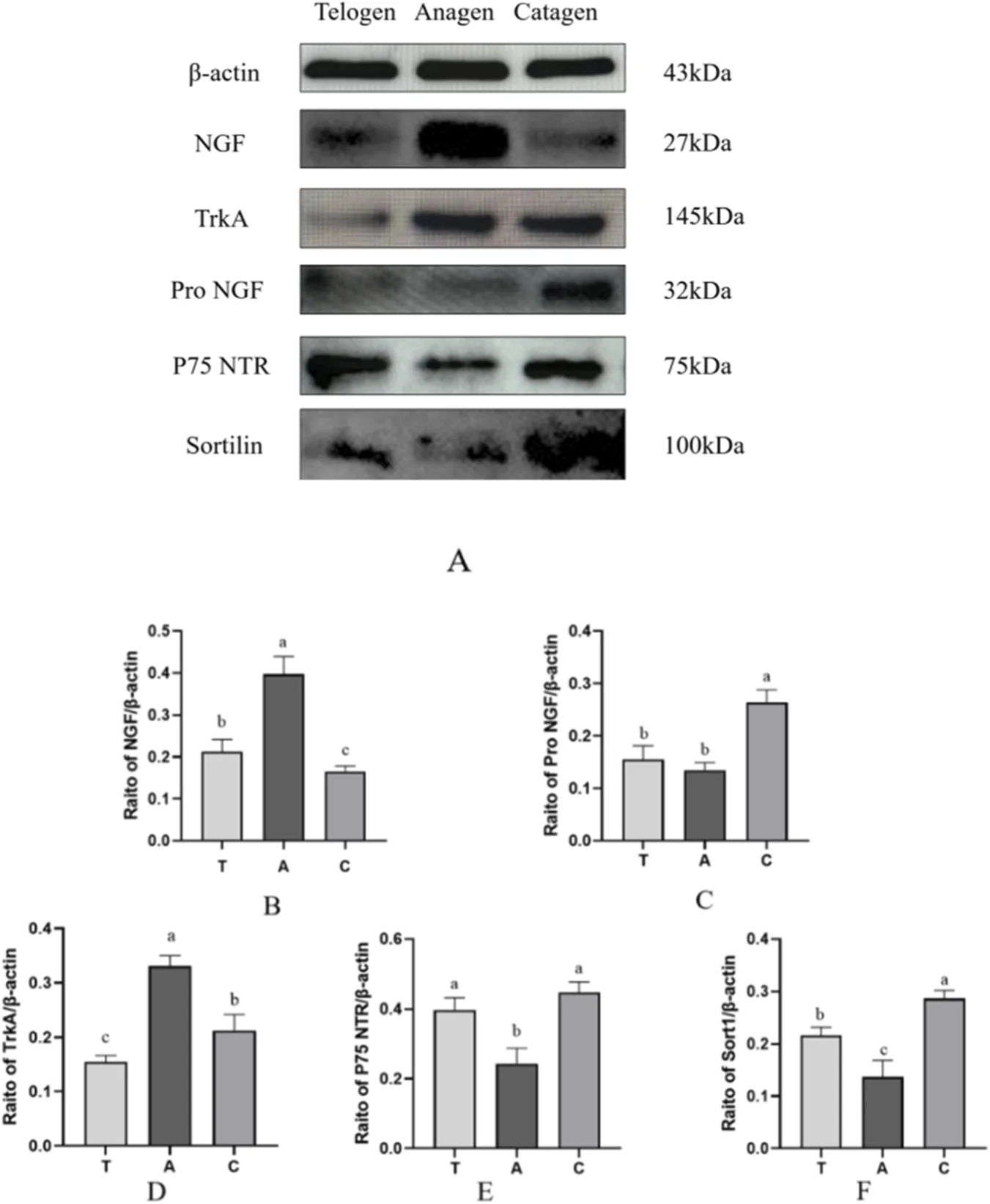
Protein expression of NGF, proNGF, TrkA, p75NTR, and Sortilin in cashmere goat skin. Note: **(A)** Representative Western blot images of each protein; **(B–F)** Quantitative analysis of protein expression levels of NGF, TrkA, proNGF, p75NTR, and Sortilin, respectively. T: telogen; A: anagen; C: catagen. Columns without common superscript letters indicate significant differences (*P* < 0.05).

### 3.4 Dynamic changes in the proNGF/NGF protein expression ratio across the hair follicle cycle

Based on the Western blot results, the protein expression ratio of proNGF to NGF was calculated for the telogen, anagen, and catagen phases of the hair follicle cycle in Liaoning cashmere goats, in order to further clarify the relative relationship between NGF and proNGF during hair follicle cycle transition. The results showed that the proNGF/NGF ratio varied across different developmental stages: the ratio was lowest in anagen (0.56), indicating that NGF predominated at the protein level; the ratio increased to 1.89 in catagen, with relatively enhanced proNGF expression; and the ratio in telogen was 1.56, falling between the two (Figure 4).

**FIGURE 4:**
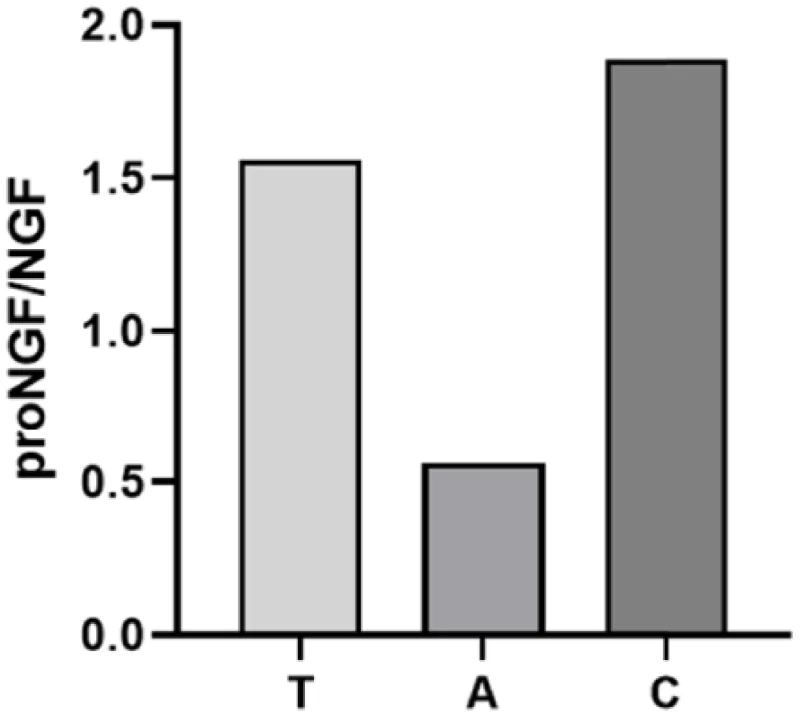
proNGF/NGF protein expression ratio across distinct phases of the hair follicle cycle in Liaoning cashmere goats. Note: T: telogen; A: anagen; C: catagen.

### 3.5 Recovery and culture of experimental cell models

Rat hair follicle stem cells (rHFSCs) and human dermal papilla cells (hDPCs) were selected as experimental models to simulate the hair follicle microenvironment *in vitro* and to investigate the functional roles of the proNGF/NGF system. After adherence, rHFSCs exhibited a relatively homogeneous morphology, predominantly polygonal or short spindle-shaped, with abundant cytoplasm and a high nuclear-to-cytoplasmic ratio. hDPCs, upon adherence, displayed a typical fibroblast-like morphology, characterized by elongated spindle shapes, aggregation, and curved, strip-like arrangements. Both cell types were cultured at 37 °C in a 5% CO_2_ incubator and proliferated stably in specialized media supplemented with 10% FBS (Figure 5). The cells exhibited favorable growth conditions, high adherence rates, and rapid recovery upon passaging.

**FIGURE 5:**
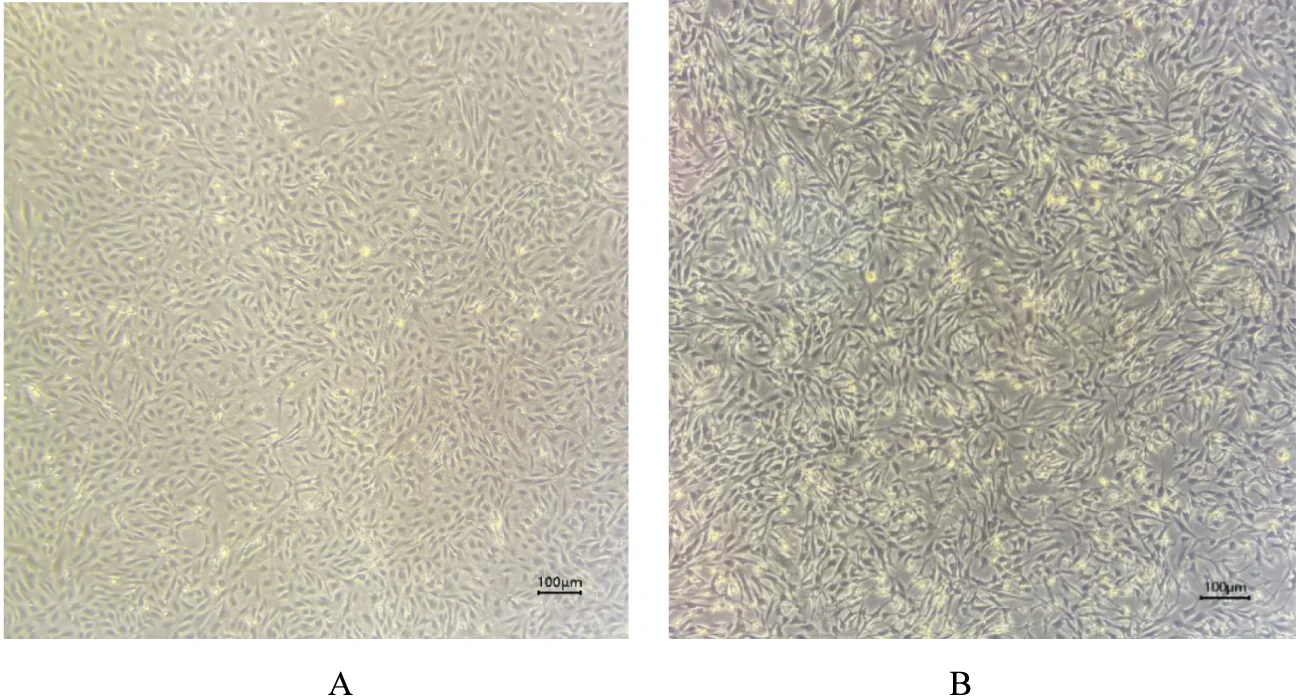
Morphology of cells cultured *in vitro*. Note: **(A)** Rat hair follicle stem cells (rHFSCs); **(B)** Human dermal papilla cells (hDPCs).

### 3.6 CCK-8 assay for cell proliferation

In the rat hair follicle stem cell model, compared with the control group, the cell proliferation rate was significantly increased in the NGF-alone treatment group (*P* < 0.05), whereas no significant difference was observed between the proNGF-alone treatment group and the control group (*P* > 0.05) (Figure 6A). In the co-treatment group with NGF and the TrkA-specific inhibitor K252a, the cell proliferation rate was significantly reduced compared with the NGF-alone group (*P* < 0.05), and showed no significant difference from the control group (*P* > 0.05) (Figure 6B). Compared with the NGF-alone group, the two mixed groups with proNGF/NGF ratios of 1.56 and 1.89 exhibited significantly decreased cell proliferation rates (*P* < 0.05), with values not significantly different from those of the control group (*P* > 0.05). However, at a proNGF/NGF ratio of 0.56, the cell proliferation rate was significantly higher than that of the control group (Figure 6A). Moreover, the proliferation rate of the mixed group with a proNGF/NGF ratio of 0.56 showed no significant difference from that of the NGF-alone group (*P* > 0.05), while remaining significantly higher than that of the control group (*P* < 0.05). As shown in Figure 6C, when the TrkA inhibitor K252a was added to the mixed-ratio groups, a significant difference was observed in the 0.56 ratio group (*P* < 0.05). Furthermore, when the proNGF/NGF ratio in the mixed groups was below 1.56, the cell proliferation rate was significantly higher than that in the corresponding K252a-supplemented groups (*P* < 0.05).

**FIGURE 6:**
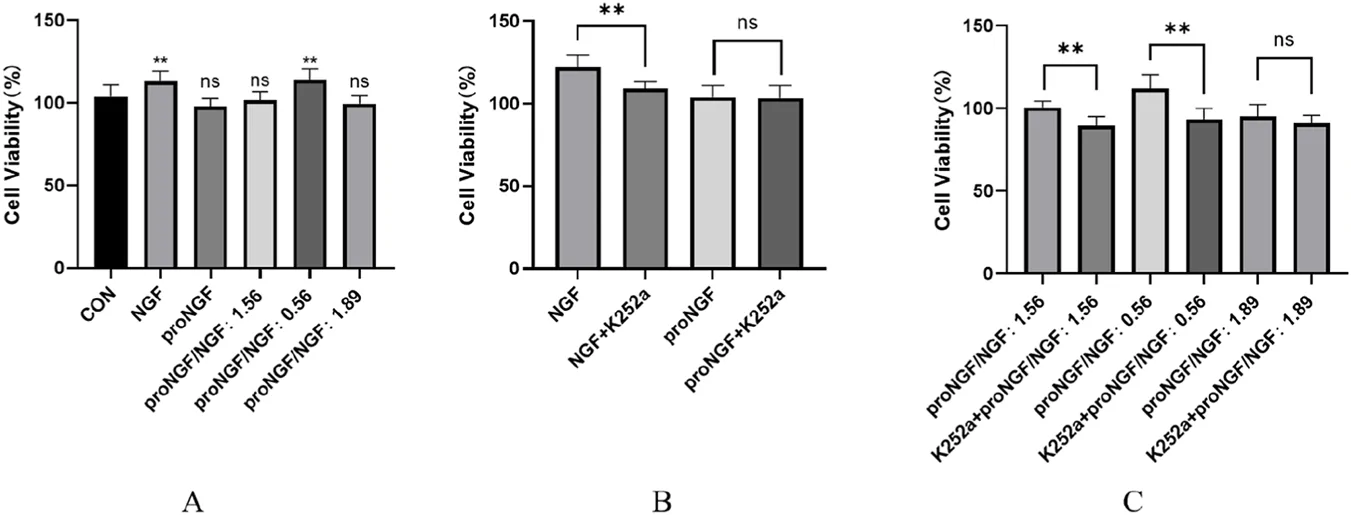
Effects of the proNGF/NGF ratio on cell proliferation rate. **(A)** Changes in cell proliferation rate in the NGF‐alone, proNGF‐alone, and mixed-ratio (proNGF/NGF) groups compared with the control (CON) group. ns, no significant difference vs. the control group; **, significant difference vs. the control group (P < 0.05). **(B)** Changes in cell proliferation rate in the NGF-alone or proNGF‐alone groups compared with the corresponding groups co-treated with the TrkA inhibitor K252a. ns, no significant difference; **, significant difference (P < 0.05). **(C)** Changes in cell proliferation rate in the mixed-ratio (proNGF/NGF) groups compared with the corresponding ratio groups co‐treated with K252a. ns, no significant difference; **, significant difference (P < 0.05).

In the human dermal papilla cell model, the results of cell proliferation were highly consistent with those observed in rHFSCs. Compared with the control group, the cell proliferation rate was significantly increased in the NGF-alone treatment group (*P* < 0.05), whereas no significant difference was detected in the proNGF-alone treatment group (*P* > 0.05). Similarly, when TrkA receptor signaling was blocked by K252a, the pro-proliferative effect of NGF was abrogated, and the cell proliferation rate in the NGF + K252a co-treatment group was significantly lower than that in the NGF-alone group (*P* < 0.05), reaching a level comparable to that of the control group (*P* > 0.05) (Figure 7). In the mixed treatment groups with proNGF/NGF ratios of 1.56 and 1.89, the cell proliferation rates were significantly reduced compared with the NGF-alone group (*P* < 0.05), and showed no significant differences from the control group (*P* > 0.05). In contrast, the mixed treatment group with a proNGF/NGF ratio of 0.56 exhibited a cell proliferation rate significantly higher than that of the control group (*P* < 0.05). To verify whether the aforementioned ratio-dependent effects were mediated through the TrkA receptor, the TrkA inhibitor K252a was added to all three mixed-ratio treatment groups. The results demonstrated that in the human dermal papilla cell model, upon the addition of the TrkA inhibitor K252a to the mixed-ratio groups, the cell proliferation rate was significantly elevated when the proportion of NGF exceeded that of proNGF (*P* < 0.05).

**FIGURE 7:**
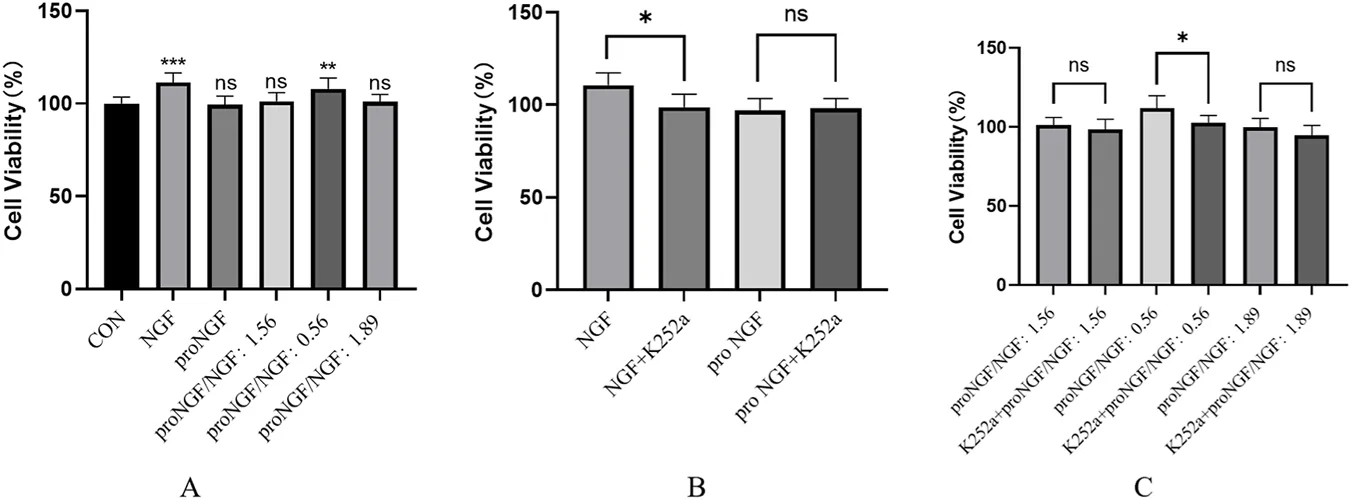
Effects of the proNGF/NGF ratio on cell apoptosis. **(A)** Changes in the apoptosis rate in the NGF‐alone, proNGF‐alone, and mixed‐ratio (proNGF/NGF) groups compared with the control (CON) group. ns, no significant difference vs. the control group; /, significant difference vs. the control group (P* < 0.05). **(B)** Changes in the apoptosis rate in the NGF‐alone or proNGF‐alone groups compared with the corresponding groups co‐treated with the TrkA inhibitor K252a. ns, no significant difference; , significant difference (P* < 0.05). **(C)** Changes in the apoptosis rate in the mixed-ratio (proNGF/NGF) groups compared with the corresponding ratio groups co-treated with K252a. ns, no significant difference; , significant difference (P* < 0.05).

### 3.7 Cell apoptosis detection

To verify the effects of NGF and proNGF on hair follicle cell apoptosis, Annexin V-PE/7-AAD double staining followed by flow cytometry was performed to assess apoptosis in rat hair follicle stem cells under different treatment conditions. A total of 11 treatment groups were established (including the control group, single-factor groups, mixed-ratio groups, and TrkA inhibitor groups), and the results are presented in Figures 8, 9.

**FIGURE 8:**
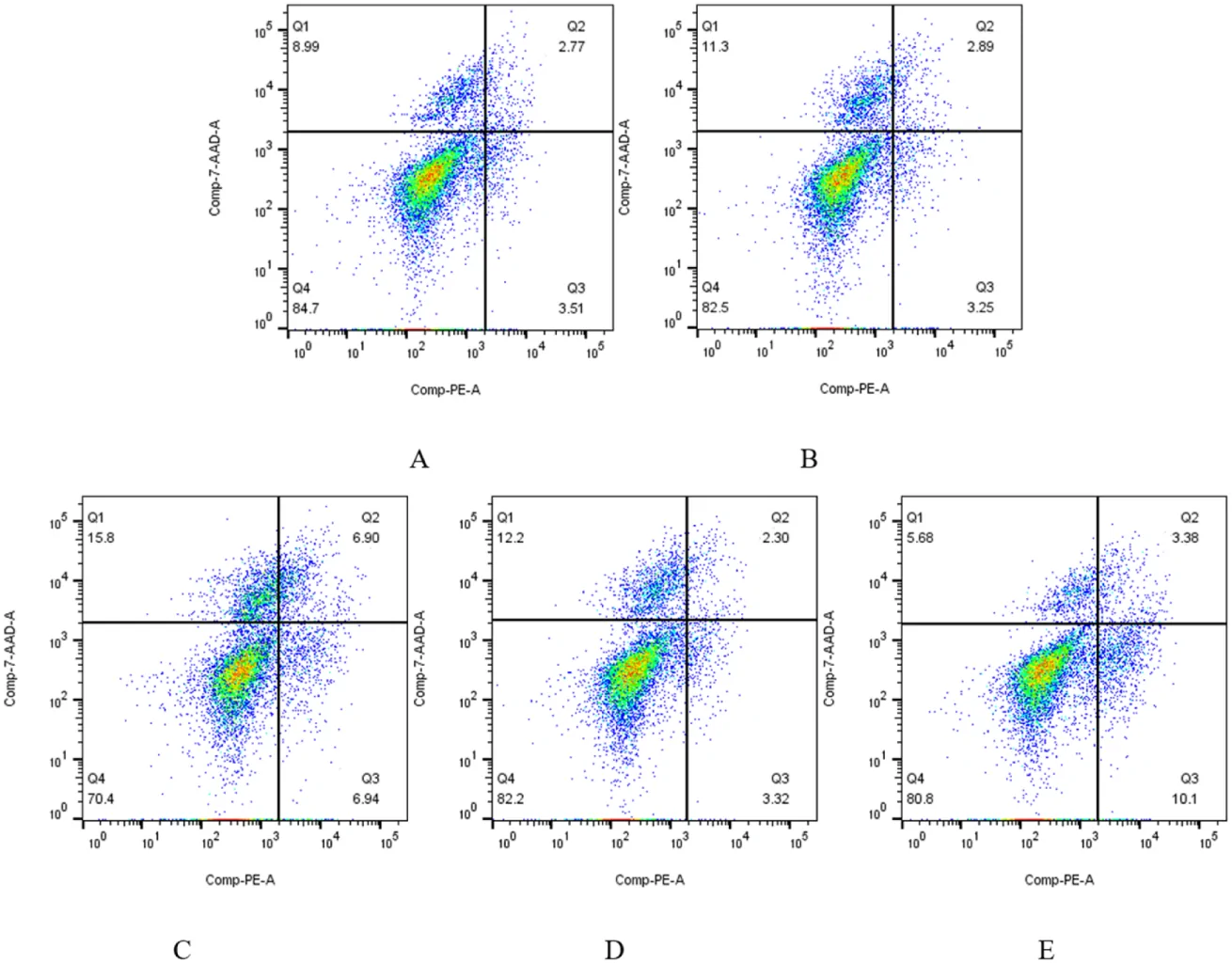
Flow cytometric analysis of apoptosis in rat hair follicle stem cells under different treatment conditions. Note: **(A)** Control group; **(B)** NGF-alone treatment group; **(C)** proNGF-alone treatment group; **(D)** NGF + K252a treatment group; **(E)** proNGF + K252a treatment group.

**FIGURE 9:**
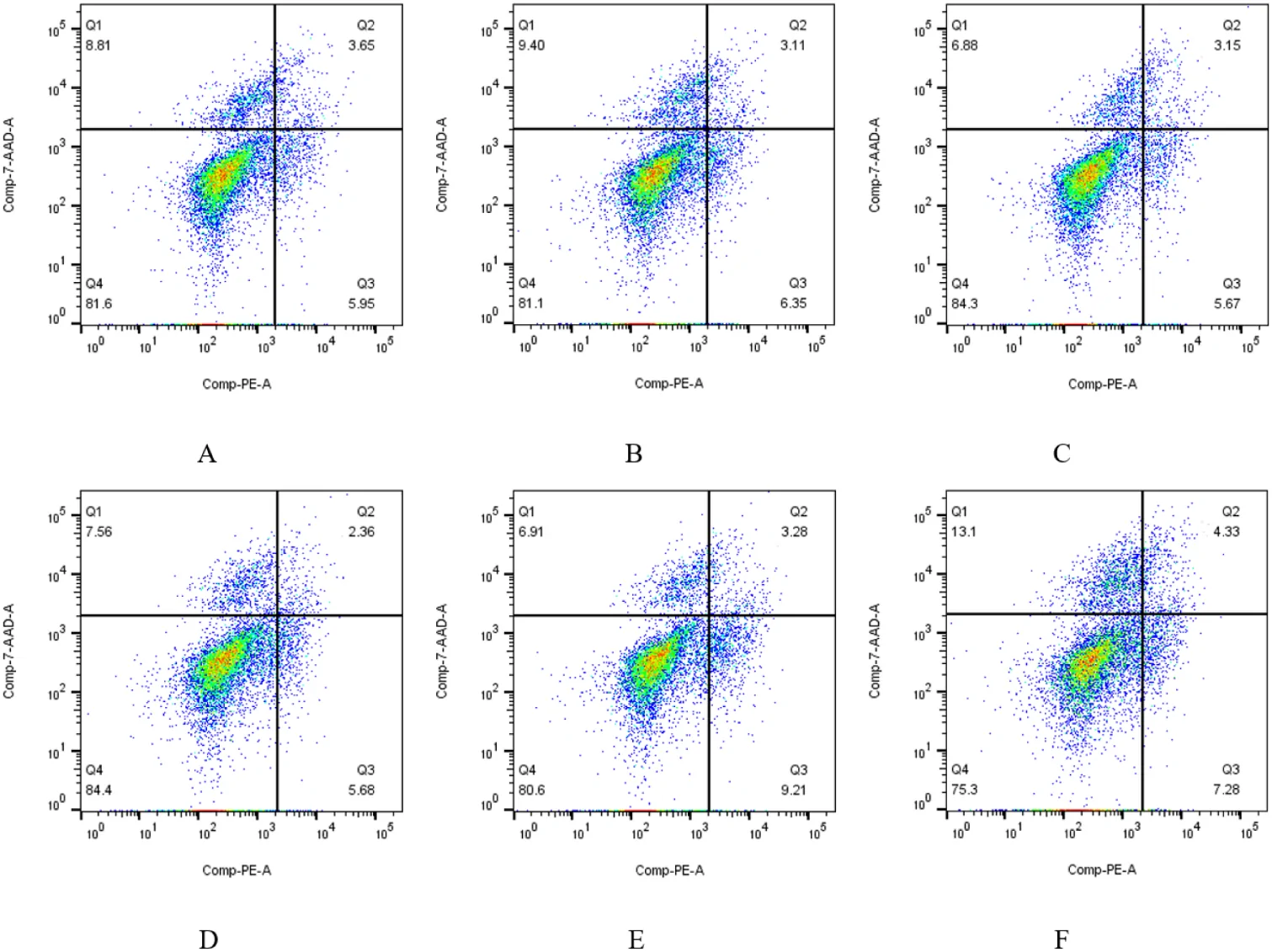
Effects of different proNGF/NGF ratios and TrkA inhibition on apoptosis in hair follicle stem cells. Note: **(A)** proNGF/NGF = 1.56; **(B)** proNGF/NGF = 1.56 + K252a; **(C)** proNGF/NGF = 0.56; **(D)** proNGF/NGF = 0.56 + K252a; **(E)** proNGF/NGF = 1.89; **(F)** proNGF/NGF = 1.89 + K252a.

Quantitative analysis of apoptosis revealed that the apoptosis rate (including both early and late apoptosis) in the proNGF-alone treatment group was higher than that in the control group, whereas the apoptosis rate in the NGF-alone treatment group showed no significant change compared with the control group, indicating that proNGF effectively induces apoptosis in hair follicle stem cells. In the NGF + proNGF mixed treatment groups, when the proNGF/NGF ratios were 1.56 and 1.89, the apoptosis rates were higher than that of the blank control group.

### 3.8 Transcriptomic sequencing

#### 3.8.1 Transcriptomic profile of hair follicle cells under different proNGF/NGF ratios

Quality assessment of the sequencing data from 12 samples revealed that the CON, NGF.H, and NGF.L groups yielded 211,792,840, 186,452,776, and 176,533,966 clean reads, respectively, after adapter trimming and low-quality read removal. The total clean data volume amounted to 83.64 Gb, with clean reads accounting for 97.9%–99.13% of raw reads. The percentage of bases with a quality score ≥30 (sequencing error rate <0.001) ranged from 94.24% to 94.91% (Table 4). The GC content ranged from 49% to 51%. Both the control and treatment groups contained four biological replicates each, and all quality control metrics were highly consistent among samples within each group, with no obvious outlier samples. These results indicate that the transcriptomic sequencing data from all samples were of high quality and sufficient for subsequent downstream analyses.

**TABLE 4:** Summary of RNA-seq data statistics.

Sample	Raw reads	Raw bases(G)	Clean reads	Clean reads ratio (%)	Clean bases(G)	Clean bases ratio (%)	Q30 (%)	GC content (%)
con1	53792638	8.07	52754896	98.07	7.64	94.67	94.50	49.68
con2	55463224	8.32	54978008	99.13	8.14	97.84	94.91	49.37
con3	61061402	9.16	60020924	98.30	8.74	95.41	94.24	49.70
con4	44910830	6.74	44039012	98.06	6.37	94.51	94.52	49.93
NGF.H1	44120222	6.62	43327742	98.20	6.31	95.32	94.39	49.63
NGF.H2	45983724	6.90	45195216	98.29	6.58	95.36	94.70	49.62
NGF.H3	53556034	8.03	52489954	98.01	7.64	95.14	94.35	49.79
NGF.H4	46403802	6.96	45439864	97.92	6.60	94.83	94.69	49.71
NGF.L1	43963238	6.59	43144938	98.14	6.28	95.30	94.45	50.26
NGF.L2	45344614	6.80	44497694	98.13	6.44	94.71	94.44	49.81
NGF.L3	45453516	6.82	44663762	98.26	6.51	95.45	94.51	49.56
NGF.L4	45177054	6.78	44227572	97.90	6.39	94.25	94.37	49.99

#### 3.8.2 Identification of key apoptosis-related differentially expressed genes

Based on the biological characteristics of the three experimental groups in this study, the numbers of significantly upregulated (log_2_FC ≥ 1 and q < 0.05) and downregulated (log_2_FC ≤ −1 and q < 0.05) differentially expressed genes (DEGs) were enumerated for each comparison. Compared with the CON group, the NGF.H group exhibited a total of 91 DEGs, comprising 62 upregulated and 29 downregulated genes. In the NGF.L group versus the CON group, a total of 94 DEGs were identified, including 40 upregulated and 54 downregulated genes. Furthermore, when comparing the NGF.L group with the NGF.H group, a total of 96 DEGs were detected, among which 28 genes were upregulated and 68 genes were downregulated (Table 5; Figure 10). Notably, the anti-apoptotic molecules Faim2 and Naip5 were significantly downregulated in this comparison.

**TABLE 5:** Differentially expressed genes among groups.

Group	Gene name	Gene description	Treatment/Control
NGF.H VS CON	*Kcna3*	Potassium voltage-gated channel subfamily a member 3	Up
	*Lcp1*	Lymphocyte cytosolic protein 1	Up
	*Lhb*	Luteinizing hormone subunit beta	Up
	*Cst7*	Cystatin F	Down
	*Dao*	D-amino-acid oxidase	Down
	*Npb*	Neuropeptide B	Down
NGF.L VS CON	*Kcna4*	Potassium voltage-gated channel subfamily a member 4	Up
	*Lcp1*	Lymphocyte cytosolic protein 1	Up
	*Lhb*	Luteinizing hormone subunit beta	Up
	*Nrsn1*	Neurensin 1	Down
	*Prdx1-ps3*	Peroxiredoxin 1, pseudogene 3	Down
	*Ramp3*	Receptor activity modifying protein 3	Down
NGF.L VS NGF.H	*Mylk4*	Myosin light chain kinase family, member 4	Up
	*Naip5*	NLR family, apoptosis inhibitory protein 5	Down
	*Mbp*	Myelin basic protein	Down
	*Faim2*	Fas apoptotic inhibitory molecule 2	Down
	*Actn3*	Actinin alpha 3	Down
	*Foxg1*	Forkhead box G1	Down

**FIGURE 10:**
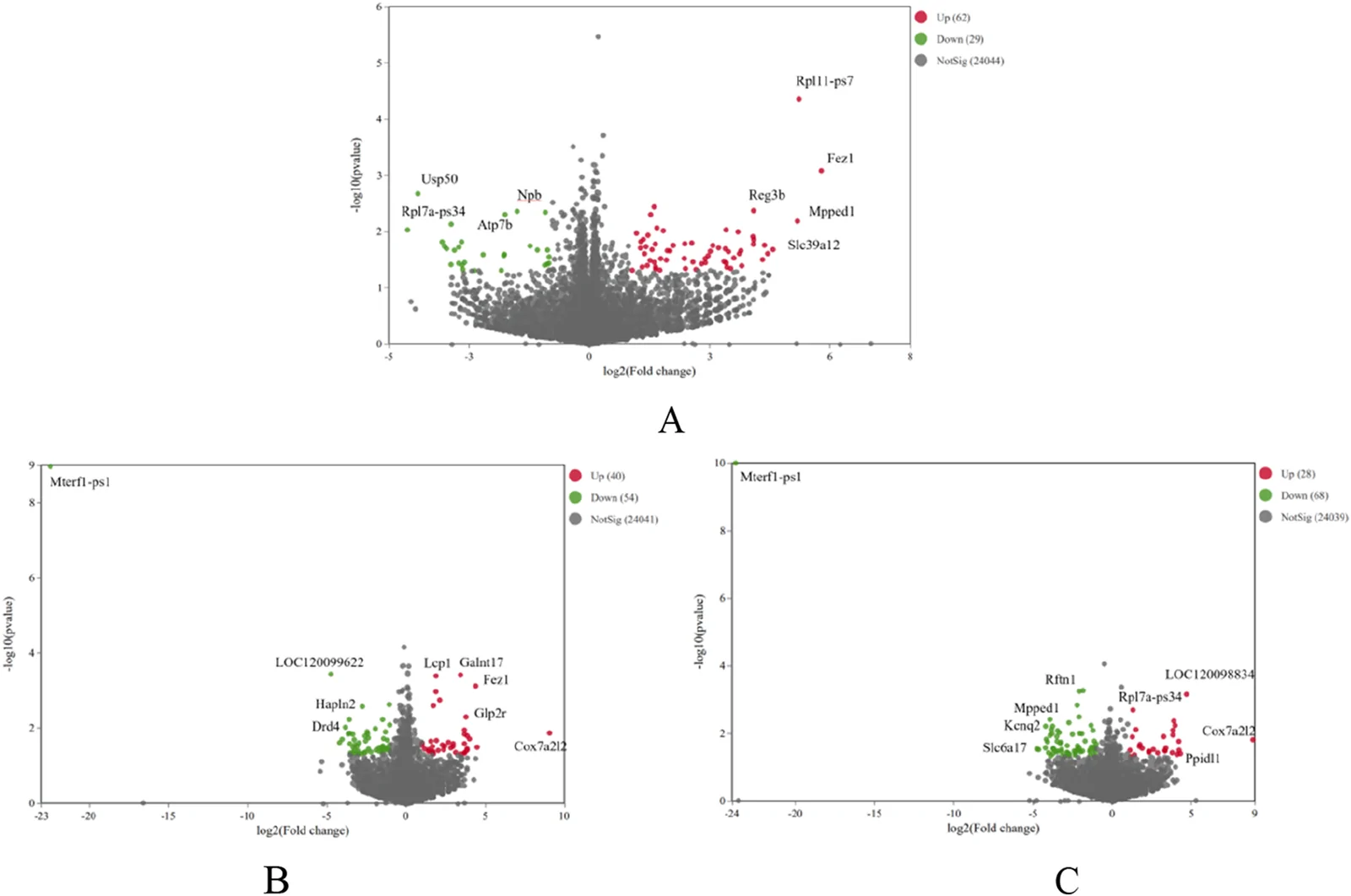
Volcano plots of differentially expressed genes among the control group and the high- and low-ratio NGF groups. Note: **(A)** Volcano plot of DEGs between the NGF.H and CON groups; **(B)** Volcano plot of DEGs between the NGF.L and CON groups; **(C)** Volcano plot of DEGs between the NGF.L and NGF.H groups.

#### 3.8.3 Functional enrichment of apoptotic and cell death pathways

In the comparison between the NGF.H group and the control group, differentially expressed genes were mainly enriched in fundamental biological categories such as “cellular process” and “metabolic process.” In the comparison between the NGF.L group and the NGF.H group, DEGs were significantly enriched in biological processes including “regulation of apoptotic process” (GO:0042981) and “cell death” (GO:0008219) (Figure 11). This enrichment was primarily driven by the downregulation of anti-apoptotic molecules such as Faim2 and Naip5, indicating that the apoptotic regulatory program is activated when proNGF predominates.

**FIGURE 11:**
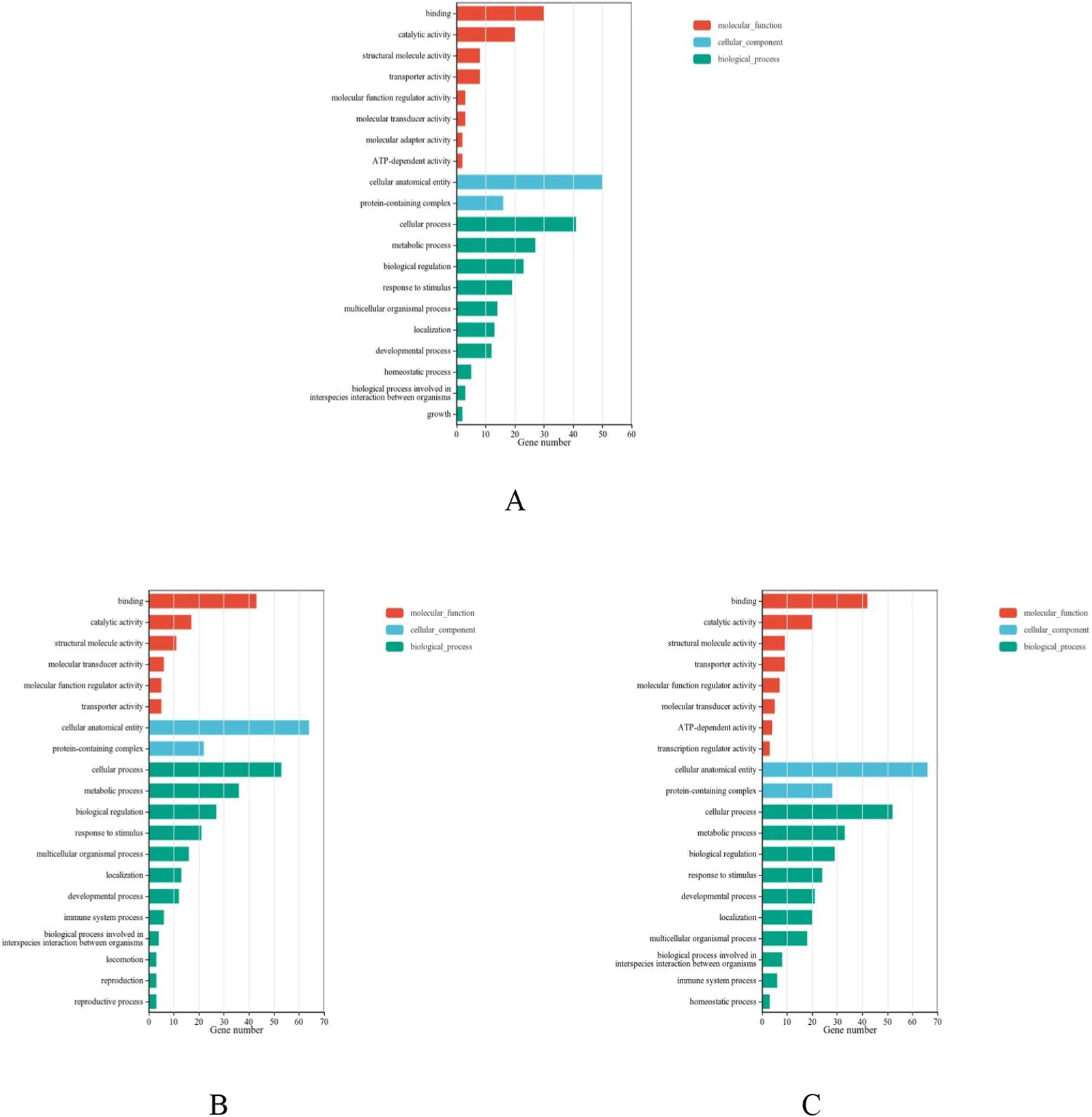
GO enrichment analysis. Note: **(A)** GO enrichment analysis between the NGF.H and CON groups; **(B)** GO enrichment analysis between the NGF.L and CON groups; **(C)** GO enrichment analysis between the NGF.L and NGF.H groups.

#### 3.8.4 Enrichment of steroid hormone and immune-related metabolic pathways

In the comparison between the NGF.H group and the control group, differentially expressed genes were significantly enriched in the Ribosome and Retinol metabolism pathways, indicating that when NGF predominates, cellular protein synthesis and metabolic activities are robust, consistent with a state of high cell proliferation.

In the comparison between the NGF.L group and the NGF.H group, DEGs were significantly enriched in the Steroid hormone biosynthesis pathway, as well as immune-related pathways such as *Salmonella* infection (Figures 12). The enrichment of the Steroid hormone biosynthesis pathway suggests that when proNGF predominates, intracellular pro-apoptotic signals such as glucocorticoids are enhanced, creating a microenvironment that inhibits growth and promotes regression, thereby driving the transition of hair follicles from the anagen to the catagen phase.

**FIGURE 12:**
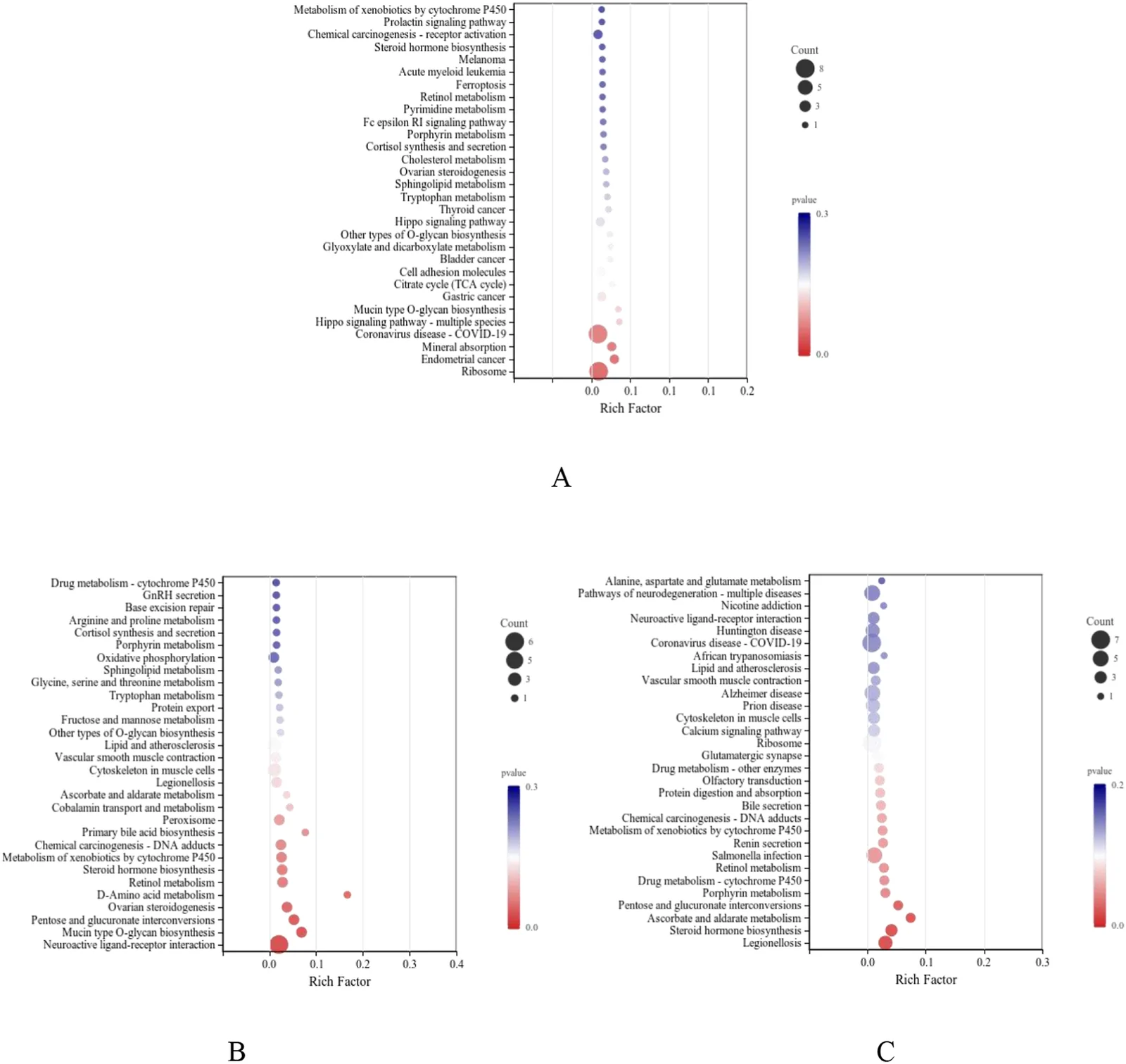
KEGG enrichment analysis. Note: **(A)** KEGG enrichment analysis between the NGF.H and CON groups; **(B)** KEGG enrichment analysis between the NGF.L and CON groups; **(C)** KEGG enrichment analysis between the NGF.L and NGF.H groups.

#### 3.8.5 Validation of transcriptomic data by quantitative real-time PCR

To validate the transcriptomic analysis results obtained from RNA-seq, seven differentially expressed genes were randomly selected for quantitative real-time PCR validation. Among them, *Abcd2, Pde1b*, and *Mylk4* were upregulated, whereas *Faim2, Actn3, Naip5,* and *Rgs7* were downregulated. Although certain quantitative differences were observed between the qRT-PCR and RNA-seq results for the seven genes, these differences were not statistically significant (*P* > 0.05), and the expression trends were consistent (Figure 13), confirming the reliability of the RNA-seq data.

**FIGURE 13:**
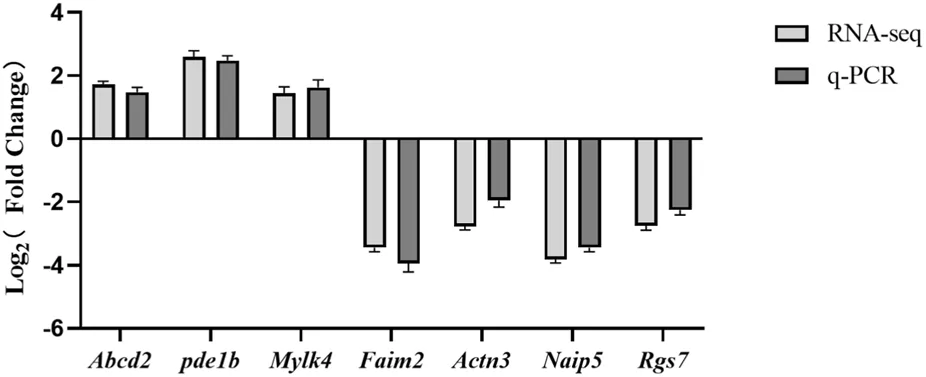
qPCR validation results.

## 4 Discussion

In this study, we systematically investigated the regulatory role of the dynamic balance of the proNGF/NGF ratio in hair follicle cycle transition in Liaoning cashmere goats, as well as the underlying molecular mechanisms. By integrating multiple lines of evidence from histology, molecular biology, cellular functional assays, and transcriptomics, we propose a “dual-system antagonism” model: during the anagen phase, the NGF/TrkA pro-survival system predominates, driving hair follicle growth and development; during the catagen phase, the proNGF/p75NTR/Sortilin pro-apoptotic system prevails, inducing follicular structural regression and cycle transition. The proNGF/NGF protein expression ratio serves a critical role as a “molecular switch” in this process, with its numerical variation reflecting the relative activity balance between the two antagonistic systems.

### 4.1 Stage-specific expression patterns of NGF system components across the hair follicle cycle

H&E staining in this study confirmed that hair follicle growth in Liaoning cashmere goats exhibits distinct staging characteristics, with secondary hair follicles displaying larger volume and higher activity during the anagen phase in September. These findings are consistent with the morphological observations of the seasonal hair follicle cycle in Liaoning cashmere goats reported by Yao et al. (2013), in which the developmental status of secondary hair follicles directly determines cashmere growth efficiency (Yao et al., 2013). Quantitative analyses at both the gene and protein levels synchronously demonstrated that the NGF/TrkA pro-growth module and the proNGF/p75NTR/Sortilin pro-apoptotic module exhibited completely opposing cycle-dependent expression patterns: NGF and TrkA mRNA and protein were significantly upregulated during anagen, whereas proNGF, p75NTR, and Sortilin reached peak expression during catagen. This spatiotemporal divergence indicates that the NGF system serves as a key regulatory molecule mediating hair follicle cycle phase transition.

Mature NGF, upon binding to the TrkA receptor, activates downstream anti-apoptotic and pro-proliferative signaling, exerting growth-promoting effects in skin hair follicles and epidermal stem cells. Previous studies have confirmed that the NGF-TrkA pathway promotes the proliferation of outer root sheath cells in cashmere goats, which corroborates the high NGF expression observed during anagen in the present study (Wang et al., 2019). In contrast, proNGF, as an immature precursor protein, lacks the ability to activate TrkA; instead, it cooperatively binds to p75NTR in conjunction with Sortilin, forming a pro-apoptotic ternary complex that initiates the mitochondrial apoptotic pathway, inducing cell shrinkage and cell cycle arrest. The synchronous high expression of proNGF and its dual receptors during catagen in our experiments suggests that proNGF predominates in the follicular microenvironment during this phase, continuously inducing apoptosis in hair follicle cells and driving follicular bulb regression and follicular degeneration. During telogen, both classes of molecules exhibited moderate expression, with the proNGF/NGF ratio falling between that of anagen and catagen, corresponding to a quiescent follicular state in which pro-survival and pro-apoptotic signals maintain a weak balance, thereby reserving stem cells for the subsequent cycle of follicular regeneration.

In this study, we quantified the proNGF/NGF protein ratios across the three phases: 0.56 during anagen (NGF-dominant), 1.56 during telogen, and 1.89 during catagen (proNGF-dominant). This graded variation in the ratio directly corresponds to the three distinct physiological states of hair follicles—growth, quiescence, and regression—demonstrating that the proNGF/NGF ratio, rather than the absolute expression level of either single molecule, serves as the core determinant of hair follicle cell fate. The exclusive detection of NGF or proNGF alone cannot comprehensively reflect the follicular growth status; rather, their relative ratio represents a molecular signature of hair follicle cycle transition. This finding constitutes the core discovery of the present study, distinguishing it from previous investigations that focused solely on the functional roles of individual NGF molecules.

### 4.2 The proNGF/NGF ratio bidirectionally regulates hair follicle cell proliferation and apoptosis via the TrkA receptor pathway

The results obtained from the two *in vitro* cell models—rHFSCs and hDPCs—were highly consistent, excluding cell-type-specific interference and robustly validating the unified regulatory effects of the NGF system on both follicular epithelial and mesenchymal cells. The CCK-8 proliferation assay demonstrated that NGF treatment alone significantly enhanced cell proliferation, whereas proNGF treatment alone exerted no pro-proliferative effect. Upon the addition of the TrkA-specific inhibitor K252a, the NGF-induced proliferative effect was completely abolished, indicating that the pro-growth function of NGF is strictly dependent on TrkA receptor signaling. In the absence of TrkA-mediated signaling, NGF failed to modulate hair follicle cell activity, which is consistent with the classical NGF-TrkA pathway mechanism established in neuronal cells (O-Sullivan et al., 2022).

A significant graded effect was observed across the different proNGF/NGF ratio treatment groups: the low-ratio group (0.56, NGF-predominant) exhibited proliferative activity comparable to that of the NGF-alone group and significantly higher than that of the control group; the medium- and high-ratio groups (1.56 and 1.89, proNGF-predominant) displayed significantly reduced proliferative capacity, reverting to levels comparable to the blank control. Flow cytometric apoptosis analysis further elucidated this phenomenon: proNGF treatment alone significantly increased the total apoptosis rate in hair follicle stem cells, whereas NGF treatment alone did not alter the apoptotic level. In the mixed-ratio systems, an increased proportion of proNGF counteracted the survival-protective effect of NGF and induced elevated apoptosis. These results indicate a competitive antagonistic relationship between proNGF and NGF: the two molecules compete for receptor binding sites within the cellular microenvironment—NGF preferentially binds to TrkA to transduce proliferative signals, whereas proNGF preferentially recruits the Sortilin-p75NTR complex to initiate apoptosis. When the relative abundance of proNGF exceeds the critical threshold of NGF (ratio >1.56), pro-apoptotic signals predominate, leading to proliferative arrest and apoptosis activation, thereby recapitulating the physiological characteristics of hair follicles during the catagen phase *in vivo*.

Upon the addition of K252a to all ratio-combination groups, the pro-proliferative effect in the low-ratio group was completely eliminated, confirming that the ratio-dependent regulatory function is fully mediated through the TrkA pathway. Based on these findings, we propose a regulatory model: when the cutaneous microenvironment exhibits a proNGF/NGF ratio <0.56, the TrkA pathway is persistently activated, driving rapid proliferation of hair follicle stem cells and dermal papilla cells to sustain the anagen phase; when the ratio rises above 1.56, the p75NTR/Sortilin apoptotic pathway prevails, resulting in extensive cell apoptosis and follicular entry into the catagen phase; at the telogen ratio of 1.56, proliferative and apoptotic signals are balanced, and follicular growth is arrested. This *in vitro* cellular model successfully recapitulates the molecular regulatory logic of the hair follicle cycle *in vivo,* confirming that the proNGF/NGF ratio functions as a functional switch governing hair follicle cycle transition.

### 4.3 Transcriptomic dissection of the downstream molecular network mediating hair follicle cell fate regulation by the proNGF/NGF ratio

In this study, RNA-seq was performed on three selected groups representing the anagen microenvironment (NGF.H, 0.56), the catagen microenvironment (NGF.L, 1.89), and the blank control group. A total of 281 differentially expressed genes were identified across the three pairwise comparisons. In the NGF.L vs. NGF.H comparison, 96 DEGs were detected, among which the anti-apoptotic genes Faim2 and Naip5 were significantly downregulated, serving as core molecular drivers of the apoptotic phenotype. GO enrichment analysis revealed that DEGs in the low-ratio NGF group (proNGF-predominant) were significantly enriched in biological processes related to regulation of apoptosis and cell death, directly elucidating the molecular basis by which a high proNGF/NGF ratio induces hair follicle cell apoptosis. Faim2 and Naip5 are classical anti-apoptotic genes: Faim2 blocks the extrinsic apoptotic pathway by interfering with the formation of the Fas death receptor complex, whereas Naip5 inhibits the intrinsic apoptotic pathway by suppressing caspase activation. The concurrent downregulation of both genes implies that cells simultaneously lose inhibition of both extrinsic and intrinsic apoptotic pathways, thereby creating conditions conducive to apoptosis initiation (Paidimuddala et al., 2023; de Lima et al., 2023; Reich et al., 2011) and amplifying the pro-apoptotic effect of proNGF. These findings are corroborated by the quantitative flow cytometric apoptosis results. In the NGF.H group, DEGs were enriched in Ribosome and basal metabolic pathways; activation of the Ribosome pathway signifies robust protein synthesis, corresponding to a physiological state of rapid cell proliferation and consistent with the high metabolic and proliferative characteristics of anagen hair follicles.

KEGG pathway enrichment analysis demonstrated that the proNGF-predominant group was significantly enriched in the Steroid hormone biosynthesis pathway. Steroid hormones such as glucocorticoids are well-established inducers of hair follicle regression, capable of inhibiting hair follicle stem cell proliferation and accelerating follicular bulb apoptosis (Kwack et al., 2017; Kwack et al., 2022). This suggests that a high proNGF/NGF ratio may upregulate the steroid synthesis pathway, thereby establishing a microenvironment that suppresses growth and promotes degeneration, synergistically driving the hair follicle cycle transition from anagen to catagen at the metabolic level. Additionally, enrichment of immune-related pathways suggests that the NGF system may indirectly modulate hair follicle development through the immune homeostasis of the skin microenvironment, providing a direction for future investigations into the interplay between skin immunity and hair follicle biology.

Seven randomly selected DEGs were subjected to qPCR validation, and the expression trends were entirely consistent with the transcriptomic data, confirming the reliability of the RNA-seq-derived DEG screening results. The apoptosis-regulatory genes and metabolic pathways identified in this study may serve as candidate marker genes for future breeding programs aimed at cashmere follicle development in cashmere goats.

### 4.4 Comparison and integration with other studies

Placing the findings of the present study within the broader literature context reveals a conserved regulatory paradigm across species. In mice, Peters et al. demonstrated that NGF/TrkA promotes anagen progression whereas proNGF/p75 facilitates catagen advancement. In human hair follicles, proNGF/NGF acts synergistically with TGF-β2 to terminate anagen via p75NTR. In cashmere goats, NGF and TrkA are highly expressed during anagen. Building upon these previous investigations, the present study makes three significant advances: first, it systematically examines the expression of proNGF and Sortilin in cashmere goats for the first time, thereby completing the comprehensive expression profile of all NGF system components across the hair follicle cycle; second, it proposes for the first time the concept of the proNGF/NGF ratio as a “molecular pointer” of hair follicle cycle status, which is validated through *in vitro* functional experiments; and third, it reveals, for the first time via transcriptomic sequencing, the regulation of downstream apoptosis-related gene networks by alterations in the proNGF/NGF ratio.

The results of this study also engage in meaningful dialogue with other signaling pathways involved in hair follicle cycle regulation. It is well established that members of the TGF-β superfamily play critical roles during the catagen phase (Song et al., 2019; Mesa et al., 2015). The enrichment of the Steroid hormone biosynthesis pathway in the present study suggests that proNGF/p75NTR signaling may cross-talk with hormonal signaling pathways. Moreover, as a member of the TNF receptor superfamily, p75NTR activation may synergize with other TNF family ligands (such as FasL and TRAIL) to collectively promote catagen progression (Chiavacci et al., 2025; Chan, 2021; Gao et al., 2017). The downregulation of Faim2 precisely provides a molecular basis for this synergistic effect—when the “brake” on the Fas pathway (i.e., Faim2) is released, Fas-mediated apoptotic signaling can be executed more effectively.

### 4.5 Limitations and future perspectives

The present study has several limitations that should be acknowledged. First, *in vivo* experiments were conducted using skin samples collected only at three key time points, which may not fully capture the subtle dynamic changes occurring throughout the entire annual hair follicle cycle. Future studies employing monthly sampling could establish a comprehensive expression profile across the complete cycle. Second, the *in vitro* experiments were performed using rat hair follicle stem cells and human dermal papilla cells rather than primary cashmere goat follicular cells; thus, species differences may introduce minor deviations in pathway signaling. Third, the present study primarily focused on the changes in NGF system components and the proNGF/NGF ratio, without directly examining the phosphorylation dynamics of downstream signaling cascades such as MAPK/ERK, PI3K/Akt, and JNK. Fourth, although transcriptomic sequencing revealed differentially expressed genes, functional validation of these genes (e.g., via gene knockout or overexpression) was not performed. Fifth, functional validation was limited to *in vitro* exogenous protein supplementation, without *in vivo* local injection of NGF/proNGF recombinant proteins into cashmere goat skin, thereby lacking direct *in vivo* functional evidence.

Future research directions may be pursued along the following lines: (1) establishing primary cashmere goat hair follicle stem cells to repeat the ratio-intervention experiments, thereby eliminating species-related discrepancies; (2) performing *in vivo* local intradermal injections of NGF or proNGF recombinant proteins to observe changes in secondary hair follicle density and length, thereby validating the regulatory functions *in vivo*; (3) conducting targeted knockdown or overexpression of Sortilin and TrkA to delineate the complete downstream signaling cascades (e.g., Akt, Caspase-3) mediated by the dual p75NTR/TrkA receptor system; and (4) screening for proteases responsible for the proteolytic maturation of proNGF, thereby modulating the proNGF/NGF ratio at the protein processing level, with the goal of developing molecular strategies to prolong the anagen phase and improve both the yield and quality of cashmere production.

## 5 Conclusion

1. The proNGF/NGF system exhibits an antagonistic expression pattern throughout the hair follicle cycle in Liaoning cashmere goats, with its dynamic ratio variation (ranging from 0.56 to 1.89) serving as a hallmark of the transition from anagen to catagen.
2. Proliferation and apoptosis of hair follicle cells are governed by the relative proNGF/NGF ratio: when the ratio is ≥1.56, apoptosis is promoted; when the ratio is 0.56, cell proliferation is enhanced.
3. When proNGF predominates within the proNGF/NGF system, it drives the molecular switch from proliferation to apoptosis in hair follicle cells through the downregulation of anti-apoptotic molecules and the activation of pro-apoptotic pathways.

## Data Availability

The RNA‐seq data generated in this study have been deposited in the NCBI Sequence Read Archive (SRA) under BioProject accession number PRJNA1535108, BioSample accession numbers: SAMN63557774 ‐ SAMN63557783. The data is available at: https://www.ncbi.nlm.nih.gov/bioproject/PRJNA1535108. All other data supporting the conclusions of this article are included within the article and its supplementary files. Further inquiries can be directed to the corresponding author.
